# Proportion of Late Presenters to HIV Care and Barriers to Early Presentation: A Mixed-Methods Study at a Tertiary Care Hospital in Uttarakhand, India

**DOI:** 10.7759/cureus.82569

**Published:** 2025-04-19

**Authors:** Athulya V Ajith, Meenakshi Khapre, Smita Sinha, Mukesh Bairwa, Teja SRK

**Affiliations:** 1 Community Medicine and School of Public Health, Postgraduate Institute of Medical Education and Research, Chandigarh, IND; 2 Community and Family Medicine, All India Institute of Medical Sciences, Rishikesh, Rishikesh, IND; 3 Internal Medicine, All India Institute of Medical Sciences, Rishikesh, Rishikesh, IND

**Keywords:** barriers to care, cd4 t-cells, hiv aids, hiv care, late presentation

## Abstract

Introduction: Late presentation to HIV care and delay of people living with HIV (PLHIV) linkage to ART continue to be a challenge in many countries of the world, including India. It has an adverse impact on the health of the patient and the outcome of antiretroviral therapy (ART) programs. In this study, we assessed the proportion of late presenters (LPs), factors associated with late presentation among PLHIV, and barriers to early presentation for HIV care among LPs in a tertiary center in India.

Methodology: A sequential mixed-method study in which the quantitative part was conducted among 156 PLHIV receiving treatment at the ART center of All India Institute of Medical Sciences, Rishikesh, India. It was followed by a qualitative study with in-depth interviews of LPs. Continuous variables such as age and CD4 count were presented as median and interquartile range (IQR) and analyzed using the Mann-Whitney U test. Categorical variables such as gender, marital status, and probable mode of transmission, represented as frequency tables and proportions, were analyzed with Pearson’s chi-square test or Fisher's exact test. A framework analysis with the help of social cognitive theory was conducted to analyze the barriers to presenting for HIV care.

Result: The prevalence of late presentation to HIV care was 77.6%. The most probable mode of transmission was sexual contact among early and LPs. Age, marital status, and type of sexual contact were found to be associated with late presentation. The barriers to timely presentation for HIV care were identified as personal, behavioral, and environmental factors, which were interrelated.

Conclusion: Regular testing of HIV in asymptomatic high-risk groups, extensive social and behavioral change communication to improve awareness, and new initiatives to sensitize healthcare professionals to enable them to provide better HIV care are necessary for the timely identification of PLHIV.

## Introduction

The HIV epidemic has been a significant global public health issue since the 1980s, and despite advances in medicine and public health, there is still no effective vaccine or treatment to eliminate HIV once infected [1,2]. The global progress against ending the HIV epidemic is slowing rather than accelerating. A recent report by the National AIDS Control Organization, India, calls for further efforts to achieve an 80% decline in annual new infections and AIDS-related deaths from 2010 to 2026 [3].

Late presentation to HIV care and delay of people living with HIV (PLHIV) linkage to antiretroviral therapy (ART) continue to be a challenge in many countries of the world, including India [1,4-6]. Late presenter (LP) to HIV care is defined as the one who is either presenting with a CD4 count below 350 cells/mm³ or presenting with an AIDS-defining illness, regardless of the CD4 count [3]. As HIV infection targets the immune system and attacks immune cells, the CD4 cell count is typically measured to assess the status of immune function. A lower CD4 count indicates a greater degree of immune system impairment and an increased risk of opportunistic infections, AIDS-related morbidities, and death. Initiation of ART in PLHIV with decreased baseline CD4 count leads to an increased cost of care and decreased benefits of the treatment due to suboptimal immune recovery [7-10]. Late presentation of PLHIV might also lead to the transfer of HIV to other individuals during the early period of the infection, which in turn adds to new HIV cases, averting the decline in the prevalence of the disease.

Despite the availability of free ART in India since 2004, many patients continue to present late to HIV care [3-5]. The test-and-treat policy for PLHIV was also introduced in 2017. Since the introduction of the above policy, studies like the one done by Rao et al. have documented a prevalence of 75.1% among LPs in South Indians [3]. The prevalence of LPs in India has decreased over time as per the studies available from 2010 to 2018 [3,4]. Various factors, such as socio-demographic, behavioral, and psychosocial, might influence the presentation of PLHIV to HIV care. The factors associated with late presentation might vary with respect to settings from one person to another [11-14].

As per the technical report of 2021, about 65% of patients living with HIV were on ART in India. If a high prevalence of patients with delayed diagnosis persists, it may hinder the achievement of the Joint United Nations Programme on HIV/AIDS (UNAIDS) 95-95-95 target. For example, if 95% of PLHIV do not know their HIV status and 95% of patients on ART have severe immunosuppression at the time of diagnosis, achieving viral suppression becomes more challenging. In this study, we aimed to assess the proportion of LP, identify the sociodemographic and clinical factors associated with it, and identify barriers for early presentation of PLHIV undergoing treatment at the ART center in a tertiary care center in Uttarakhand. We conducted a qualitative study to identify the barriers and understand the perspective and circumstances of LP that hinder early presentation.

## Materials and methods

Study design

A sequential explanatory mixed-method study [15] was conducted at the ART Center in Uttarakhand, India. The study consisted of two components: a quantitative cross-sectional study and a descriptive qualitative study. The two methods were used sequentially, with the survey undertaken before the interviews. Both methods were combined for complementarity, where each method addressed distinct aspects of the research question and for development, where the quantitative component facilitated sampling for qualitative components.

Quantitative study

The quantitative study involved a hospital-based survey of PLHIV receiving treatment at the ART Center (November 2021 to November 2022). The study included PLHIV receiving treatment at the ART Center, All India Institute of Medical Sciences, Rishikesh, India, who were from different districts of Uttarakhand, India. Eligible participants were consecutive OPD attendees at the ART clinic. The study included both old and new PLHIV between the ages of 18 and 60. Participants who were unwilling to give consent or had cognitive impairments or known mental disorders were excluded. The sample size was estimated considering a prevalence rate of 75% LP among PLHIV from a previous study conducted in South India [3], and it was done for a confidence level of 95% and a non-response rate of 5%. Accordingly, the sample size of 156 was determined using OpenEpi version 3.1. A pretested structured questionnaire (Appendix A) was administered, which included sociodemographic information, HIV status of the spouse, probable mode of transmission, CD4 count, and clinical characteristics at the time of diagnosis.

Qualitative study

For the qualitative part of the study, purposive sampling was used to select participants who were identified as LPs in the earlier phase. Data was collected through in-depth interviews using a predetermined interview guide (Appendix B). The guide included questions about participants' knowledge of HIV, reasons for getting tested, support from friends and family, and difficulties in taking ART. The interviews were conducted by a trained qualitative researcher (AV) and lasted between 45 minutes and 1 hour. Interviews were scheduled as per the convenience of the participants and continued till data saturation.

Data management and analysis

Quantitative Part

Data from the quantitative part was entered in MS Excel version 2302 (Microsoft Corporation, Redmond, USA) and analyzed using IBM SPSS Statistics for Windows, Version 25 (Released 2017; IBM Corp., Armonk, New York, United States). Data was presented in terms of the median and interquartile range (IQR) for continuous variables such as age and CD4 count, as it was not normally distributed on applying the Shapiro-Wilk test, and analyzed using the Mann-Whitney U test. Categorical variables such as gender, occupation, marital status, and probable mode of transmission were represented as frequency tables and proportions, and the difference between the groups was analyzed with Pearson’s chi-square test or Fisher's exact test. A p-value less than 0.05 was considered significant.

The quantitative result was related to the qualitative part for identifying the study participants.

Qualitative Part

The qualitative part involved concurrent data collection and analysis. Following each interview, the researcher transcribed the audio recording and conducted an analysis to identify words and phrases in the local language (Hindi). The verbatim translation to English was thoroughly cross-checked to ensure accuracy and completeness. Two autonomous researchers thoroughly examined the transcripts individually to do open coding of each transcript using the trial version of MAXQDA software (VERBI Software GmbH, Berlin, Germany). Relevant and illustrative codes were compared, differences were resolved, and repetition was eliminated by discussion. The study employed a framework analysis to examine the obstacles to accessing HIV care [1]. The codes were categorized into many groups, including personal, contextual, and behavioral aspects, based on Bandura’s social cognitive theory in the context of HIV [1,2]. Within the framework, a new structure was created for the data through analyzing the interrelationship and interaction of these factors.

## Results

Quantitative part

Among the 156 study participants, 121 (77.6%) were LPs and 35 (22.4%) were early presenters (EPs). The median value of the CD4 count of study participants at the time of presentation was 199/mm^3 ^with a range of 1250 (13-1263)/mm^3^.

No significant difference was found among EP and LP with respect to gender, residence, SE status, level of education, and occupation. There was a significant difference in the current age and age at the time of diagnosis between the LPs and EPs (p < 0.05). Among the total study participants, 68 (43.6%) were employed in private/government service at the time of being diagnosed with HIV, which was also the majority in the case of LPs (55, 45.5%) and the EPs (13, 37.1%). Forty-six out of the 68 study participants were doing service in the private sector, 12 were hotel staff, and 2 were Ashrama workers. Among the people who were drivers, four were truck drivers, and three were local transport workers. Men who have sex with men (MSM), transgender, and intravenous drug users were mostly EP, while migrants and truck drivers were LP (Table 1).

**Table 1:** Characteristics of socio-demographic and partner profile of study participants Chi-square analysis/chi-square statistic with Yates correction or ^$^Fisher Exact test was performed between study groups for all qualitative variables except age, for which ^#^Mann-Whitney U test was used. ^z ^p-value could not be calculated as some of the cells have 0 value. LP: late presenter; EP: early presenter

Characteristics	Total (N = 156)	LP (N = 121)	EP (N = 35)	Value of test of significance	p-value
Age (years) median (IQR)	40 (15)	41 (14)	32 (16)	1439	0.004^#^
Age at the time of diagnosis (years) median (IQR)	37 (17)	37 (15)	29 (16)	1597.5	0.027^#^
Gender, n (%)	Total (N = 156)	LP (N = 121)	EP (N = 35)		
Male	86 (55.1)	70 (57.9)	16 (45.7)	0.922	0.337
Female	68 (43.6)	51 (42.1)	17 (48.6)		
TG	2 (1.3)	0 (0)	2 (5.7)		(-)^z^
Residence, n (%)	Total (N = 156)	LP (N = 121)	EP (N = 35)		
Rural	127 (81.4)	96 (79.3)	31 (88.6)	0.98	0.32^$^
Urban	29 (18.6)	25 (20.7)	4 (11.4)		
Socio-economic status (as per modified BG Prasad classification 2021), n (%)	Total (N = 156)	LP (N = 121)	EP (N = 35)		
Upper	10 (6.4)	8 (6.6)	2 (5.7)	2.324	0.689
Upper middle	34 (21.8)	26 (21.5)	8 (22.9)		
Lower middle	37 (23.7)	27 (22.3)	10 (28.6)		
Upper lower	55 (35.3)	42 (34.7)	13 (37.1)		
Lower	20 (12.8)	18 (14.9)	2 (5.7)		
Level of education, n (%)	Total (N = 156)	LP (N = 121)	EP (N = 35)		
Illiterate	31 (19.9)	25 (20.7)	6 (17.1)	4.574	0.469
Primary	30 (19.2)	24 (19.8)	6 (17.1)		
Secondary	21 (13.5)	15 (12.4)	6 (17.1)		
High school	36 (23.1)	30 (24.8)	6 (17.1)		
Intermediate	14 (9)	8 (6.6)	6 (17.1)		
College and above	24 (15.4)	19 (15.7)	5 (14.3)		
Occupation, n (%)	Total (N = 156)	LP (N = 121)	EP (N = 35)		
Private/government service	68 (43.6)	55 (45.5)	13 (37.1)	0.809	0.667
Housewife	47 (30.1)	35 (28.9)	12 (34.3)		
Self-employed	32 (20.5)	24 (19.8)	8 (22.9)		
Driver	7 (4.5)	7 (5.8)	0 (0)		
Student	2 (1.3)	0 (0)	2 (5.7)		
Marital status, n (%)	Total (N = 156)	LP (N = 121)	EP (N = 35)		
Single	21 (13.5)	11 (9.1)	10 (28.6)	8.859	0.012
Married	104 (66.7)	85 (70.2)	19 (54.3)		
Widow/widower	31 (19.9)	25 (20.7)	6 (17.1)		
HIV status of spouse, if partner alive, n (%)	Total (N = 104)	LP (N = 85)	EP (N = 19)		
Positive	68 (65.4)	55 (64.7)	13 (68.4)	2.805	0.246
Negative	23 (22.1)	21 (24.7)	2 (10.5)		
Unknown	13 (12.5)	9 (10.6)	4 (21.1)		
HIV status of spouse if widowed, n (%)	Total (N = 31)	LP (N = 26)	EP (N = 5)		
Positive	17 (54.8)	14 (53.8)	3 (60)	0.056	0.812
Unknown	14 (45.2)	12 (46.2)	2 (40)		
History of Tattooing, n (%)	Total (N = 156)	LP (N = 121)	EP (N = 35)		
Yes	25 (16.0)	20 (16.5)	5 (14.3)	0.102	0.75
High risk or bridge population, n (%)	Total (N = 14)	LP (N = 9)	EP (N = 5)		(-)^z^
Intravenous drug users	3 (21.4)	2 (22.2)	1 (20)		
Migrant	2 (14.3)	2 (22.2)	0 (0)		
Men who have sex with men (MSM)	3 (21.4)	1 (11.1)	2 (40)		
Transgender	2 (14.3)	0 (0)	2 (40)		
Truck driver	4 (28.6)	4 (44.4)	0 (0)		

The majority of the study participants were married (104, 66.7%) at the time of diagnosis of HIV. There was a significantly higher proportion of married people in LP compared to EP. No significant difference between EP and LP in known HIV status of the spouse and history of tattooing (Table 1).

**Figure 1:**
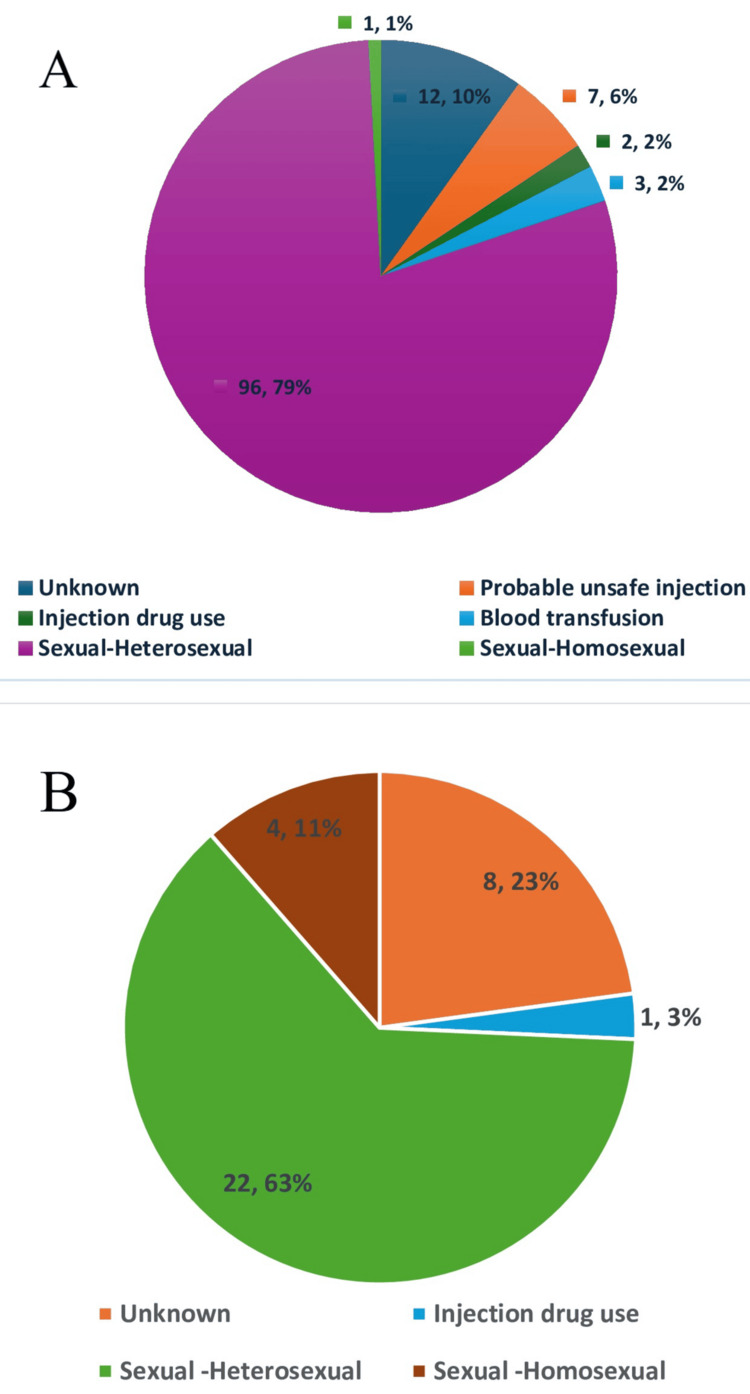
Probable mode of transmission among late presenters and early presenters (A) Probable mode of transmission among late presenters; (B) Probable mode of transmission among early presenters

**Figure 2:**
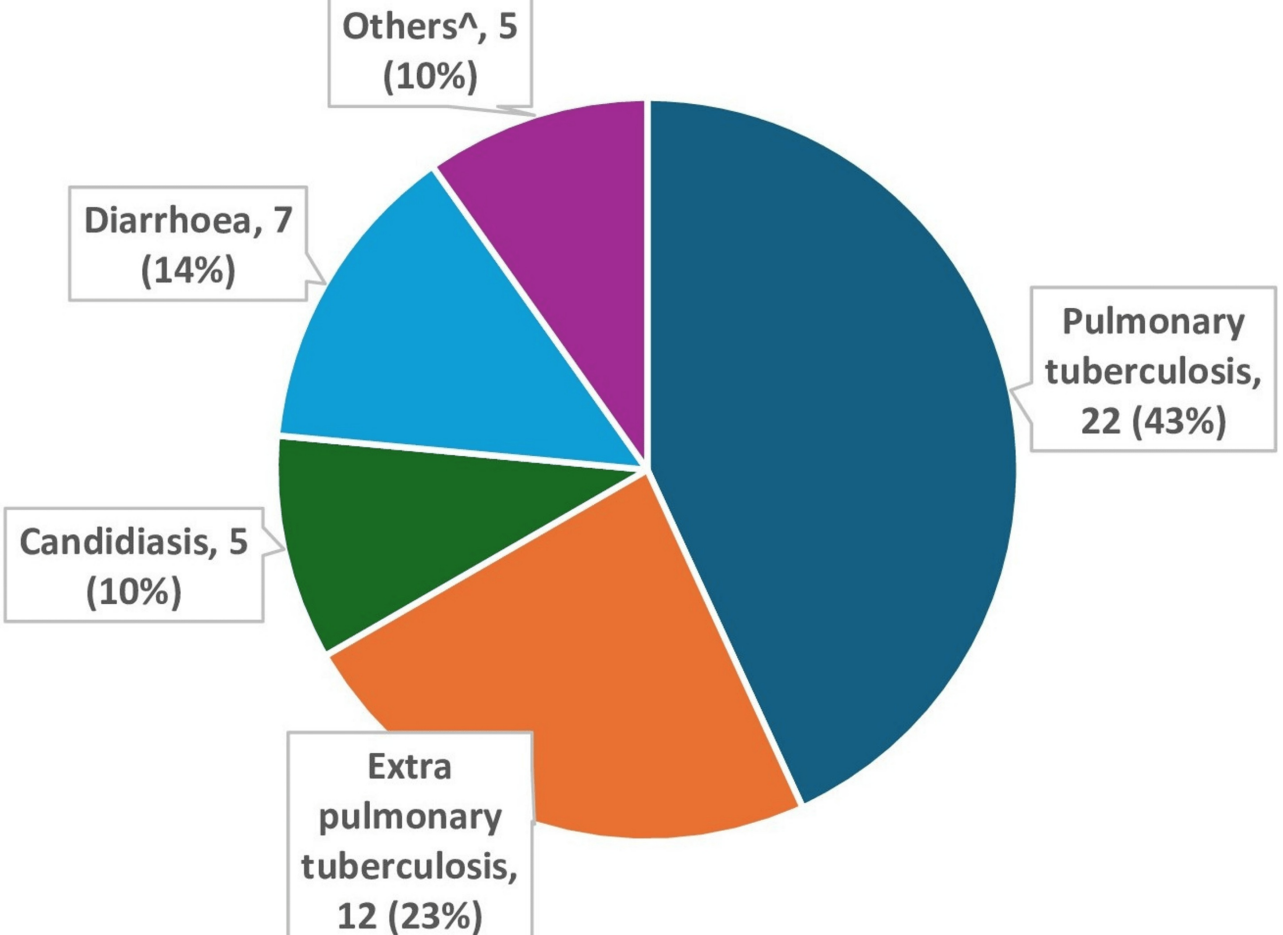
Opportunistic infections among the total study participants ^Others include one case each of pneumocystis pneumonia, toxoplasmosis, onchocerciasis, pelvic inflammatory disease (PID), and scabies.

The most probable mode of transmission among the study participants was sexual contact (97, 78.8%), similar in both EP and LP (Figures 1A-1B). There was no statistically significant association observed between mode of transmission and time of presentation (p = 0.131). Four out of 5 (80%) of the homosexuals were EP, and a significant difference was observed in the type of sexuality (p = 0.007) (Figure 2). Of all the participants, one healthcare worker was an LP with no history of any needle prick injury.

The reason for HIV testing was significantly different between EP and LP. Most of the LPs were referred by a physician (97, 80.2%) to get tested for HIV due to various symptoms (p = 0.002). Of the total study participants who were referred by the physician for HIV testing, most were due to the spouse/partner being diagnosed positive (37, 31%), which was similar in EP and LP. Nine out of 16 EPs who were diagnosed incidentally were diagnosed during antenatal care (ANC) check-up.

Among the total participants, 91 (58.3%) were diagnosed at WHO stage 1, with a higher proportion among EPs (32, 91.4%). There was also a significant association with the presence of opportunistic infection and the time of presentation, as only three (8.5%) EPs had opportunistic infection (p = 0.001) (Table 2).

**Table 2:** Reason for HIV testing and clinical profile A chi-square statistic with Yates correction was performed between the study groups (^a^ stages 2, 3, and 4 were combined, and ^b^ weight loss was merged with others to apply chi-square statistics).

Characteristics	Total (N = 156)	LP (N = 121)	EP (N = 35)	Value of test of significance	p-value
Reason for HIV testing/ diagnosis of HIV, n (%)					
Incidental diagnosis	40 (25.6)	24 (19.8)	16 (45.7)	9.5362	0.002
Referred by physician	116 (74.4)	97 (80.2)	19 (54.3)		
Symptoms/reason for referral to HIV testing, n (%)	Total (N = 116)	LP (N = 97)	EP (N = 19)		
Dermatological symptoms	6 (5.2)	4 (4.1)	2 (10.5)	6.71^b^	0.151
GI symptoms	18 (15.5)	17 (17.5)	1 (5.3)		
Respiratory symptoms	22 (19)	21 (21.6)	1 (5.3)		
Spouse diagnosed positive	37 (31.9)	30 (30.9)	7 (36.8)		
Weight loss	9 (7.8)	7 (7.2)	2 (10.5)		
Others	24 (20.7)	18 (18.6)	6 (31.6)		
WHO clinical stage, n (%)	Total (N = 156)	LP (N = 121)	EP (N = 35)		
Stage 1	91 (58.3)	59 (48.8)	32 (91.4)	18.617^a^	0
Stage 2	24 (15.4)	21 (17.4)	3 (8.6)		
Stage 3	26 (16.7)	26 (21.5)	0 (0)		
Stage 4	15 (9.6)	15 (12.4)	0 (0)		
Presence of opportunistic infections/comorbidities, n (%)	Total (N = 156)	LP (N = 121)	EP (N = 35)		
Yes	46 (29.5)	43 (35.5)	3 (8.5)	8.241	0.004

Five of the LPs were having more than one infection at the time of diagnosis. Pulmonary TB was the most prevalent infection, followed by extrapulmonary tuberculosis, diarrhea, and candidiasis. All individuals diagnosed with both pulmonary and extrapulmonary tuberculosis presented at a later stage (Figure 2).

Qualitative part

**Figure 3:**
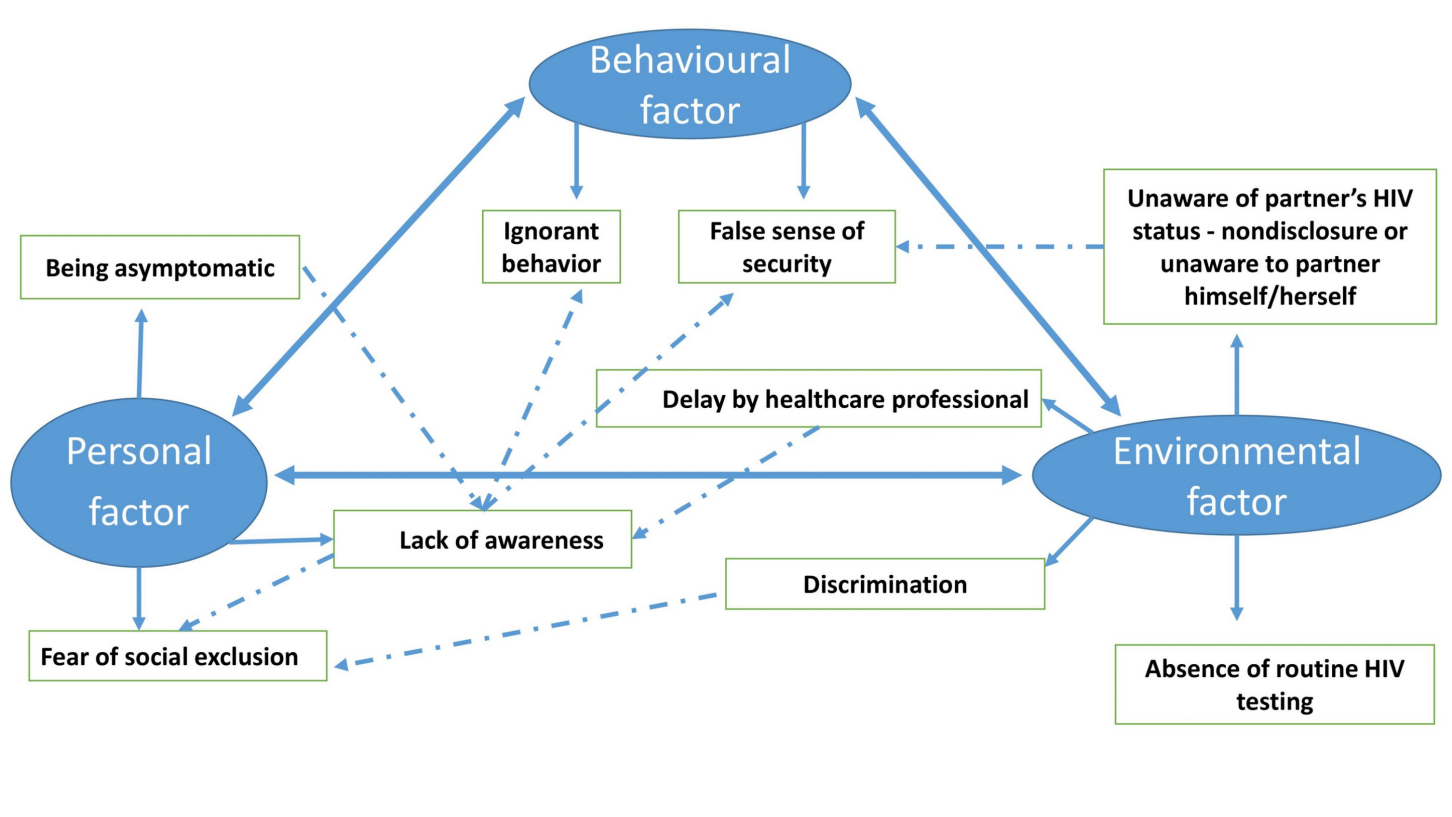
Diagrammatic representation of barriers to presenting for HIV care as per the social cognitive theory The solid-thick arrow shows the interrelation between the three main components or themes of social cognitive theory; the solid-thin arrows show the subthemes that fall under each of these themes, and the dotted arrows depict the interconnection between various subthemes. Credit: Image created by the authors

An in-depth interview was conducted among the eight LPs between 29 and 50 years of age. Half of them were males and females, respectively. Two participants had a history of blood transfusion - one during a surgical procedure and the other following a workplace injury. As per the social cognitive theory, the identified codes were categorized under personal, behavioral, and environmental/social factors (Table 3), and Figure 3 depicts the barriers to presenting for HIV care and their interrelation.

**Table 3:** Themes generated during in-depth interviews to identify barriers in seeking HIV care among late presenters

Theme	Sub theme	Code	Verbatim
Personal factors	Lack of awareness	Unawareness about the mode of spread of HIV	“In everyone’s mind sometime or the other this thought comes that is it happening due to physical relationship” (P.1/female)
		Unaware of symptoms, testing, and treatment	“I knew that when blood comes in contact with blood it happens, but I didn't know it would happen to myself” (P.5/male)
	Fear of social exclusion /loss of social support	-	“There was a lot of fear, and it seemed that life was over if it becomes positive by chance. The mind was refusing, it was saying that if it becomes positive even by mistake, then it will be very difficult” (P.6/male)
	Being asymptomatic	-	“Many people get to know in after years because their health seems good” (P.1/female). “Before this, there was no difficulty, even though awareness was there. I didn't even suffer from fever for 2-2.5 years” (P.6/male).
Behavioral factors	Ignorant behavior	-	“Yes, I knew that they had this thing, and they are taking medicines. they used to tell that you have to think about getting tested and I used to say that I do not have this thing. And later I found out that I also have” (P.3/female).
	False sense of security	-	“I have not done anything wrong like that in my life. So, I had no such thought in my brain” (P.4/male). “I was sure that it wouldn’t have it and i was like this that it would be negative... it would be negative only” (P.1/female).
Environmental/social factors	Delay by health care professional	Delay in advising test by a health care professional	“It was said that it is an infection, it is an infection, but he probably did not do the test, the virus one” (P.1/female).
		Lack of counselling by healthcare professionals	“No, no doctor or staff from here has explained how this disease spreads. Do I have to get my son also tested?" (P.8/female).
	Unaware of partner’s HIV status - nondisclosure by partner or not aware of partner	-	“He had TB. I don't know about HIV” (P.8/female).
	Discrimination	Social discrimination	“The society does this, leave this person and do not talk to him. Our society believes this, to not touch clothes, do not touch his drinks and food, keep his utensils separate. Don't keep your hands on him. Treats like an untouchable” (P.1/female).
		Gender discrimination	“There was a case near our house. So, one male had HIV, and his parents came to know that he had HIV, so they said first get your wife tested, don't know how many people our daughter-in-law has tried hitting. First of all, the thought is that it can only happen physically” (P.1/female).
	Absence of routine HIV testing	-	“As such, along with other blood tests, the one of HIV should also be there” (P.1/female) "But I believe that like a routine check-up, a person should also get this HIV test done every 6 months" (P.4/male).

## Discussion

The prevalence of late presentation to HIV care was 77.6% in the present study. The most probable mode of transmission was sexual contact in both EPs and LPs, which was around 74% and 80%, respectively. Older age, being married, and heterosexuality were identified to be correlates for late presentation. The presence of opportunistic infections and being referred by a physician for HIV testing was significantly higher among the LP. In assessing the barriers to presentation to HIV care with respect to social cognitive theory, we identified interrelated themes with respect to personal, behavioral, and environmental factors.

Our findings, prevalence of late presentation (77.6%), were consistent with other studies from different parts of India, such as Andhra Pradesh, Delhi, and Karnataka [3-5]. We could not find any other study in Indian settings after 2018. A high prevalence of LPs to HIV care was observed in various studies from African countries, despite a declining trend found in certain recent studies [6,16-18]. As per the cross-European update, no decrease in LP was found from 2010 to 2013, with a 48.7% prevalence of LP in the year 2013 [14]. A cohort study of the Asia-Pacific region showed a prevalence of 72%, which included a mix of low and high-income countries [13]. A high prevalence of LPs was observed in almost all the studies, and the trend shows a decrease in countries where initiatives for routine testing were introduced. Studies from developing regions such as sub-Saharan Africa, Southeast Asia, and South America reported that the late presentation was due to limited healthcare access and health literacy [13,18,19]. The reasons behind this high prevalence of late presentation might be a lack of awareness, absence of routine testing for HIV, and delayed identification of early symptoms by health care professionals, as confirmed by in-depth interviews.

Like results from other studies [3,4,6,12,20], the median age of LP at the time of diagnosis is significantly higher than that of EP. There are chances of HIV infection being recent at a young age, whereas at an older age, the longer duration of disease is causing further immunosuppression and decreased CD4 count. Among the older age group, it might also be relatively less self-perceived risk due to a lack of awareness or missed opportunities in the clinical settings [3].

No significant association of gender was observed, though males were slightly more than females among LP. Both the transgender participants in the study were EP, and the reason might be that they are the target group for HIV testing by non-governmental organizations and others. Studies showing a significant association between the male gender and late presentation explained it due to a lack of male-oriented HIV control strategies and plans, while early diagnosis in females may be due to routine checking during antenatal visits and measures to prevent mother-to-child transmission. [3,4,7,13,14,18]. In the present study, all the females who were diagnosed during routine ANC check-ups were EP, and the results align in line with these studies.

All participants who were drivers by occupation were LPs. Unexpectedly, we found hotel workers constituted a good proportion of private service jobs (8%), reminding us to pay attention to various occupational groups among migrant workers staying away from their families. Among housewives, 74.4% were LPs. They were mostly diagnosed after the partner had complications of HIV or at the time of the partner’s death. A significant association was seen between marital status and LP, as another study concluded that being single was a protective factor for late presentation [8]. The probable reason for the observed result is that single individuals were of a younger age and had better knowledge about their exposure and associated risk.

The significant association of WHO clinical stage with late presentation replicated results from earlier studies stating that, along with a fall in CD4 count, further immunosuppression occurs, which leads to increased chances of patients presenting in advanced stages of HIV [17]. Opportunistic infections/comorbidities at the time of diagnosis were significantly associated with late presentation, and the findings are in line with the previous findings of studies conducted in Ethiopia [9,10] and India [21,22]. In our study, pulmonary tuberculosis was the most common opportunistic infection, followed by extrapulmonary tuberculosis, candidiasis, and diarrhea. Tuberculosis is the most reported opportunistic infection associated with HIV in India for years [23], and initiatives of routine HIV testing among tuberculosis patients and tuberculosis preventive therapy must be emphasized further.

Most of the participants in the study had sexual contact as a probable mode of transmission, which was similar in EP and LP. Like other studies from India [3,4], Eastern Sicily [24], Belgium [25], and Switzerland [20], heterosexual contact was associated with late presentation. The better perception of risk among homosexuals might be the influencing factor for getting tested and being diagnosed at an early stage [4,25]. Contrary to the assumption, we found that the general population is at a higher risk of being LPs compared to the high or bridge population. Unlike a few other studies [4,5], injecting drug users (IDUs) were not associated with late presentation, possibly due to the small size of this group in our study or the identification of IDUs as a high-risk group being focused on for HIV testing and awareness sessions [25]. All the participants who had a blood transfusion as the probable mode of transmission were identified as LP. Absence of any routine testing in such patients at least once after blood transfusion, through which high viral load exposure happens at a single point in time, can be a possible reason [26].

Barriers to seeking HIV care are usually borne out of individual behavior and beliefs, whilst environmental factors can have either a positive or negative influence on taking the test [27]. A key issue behind the late presentation was the unawareness about the mode of spread, symptoms, testing, and treatment of HIV/AIDS, which influences an individual’s perception of the risk of this disease, as observed by various other studies as well [11,27,28].

Social stigma and discrimination are well documented in existing literature as barriers [27,29,30] to seeking HIV care and are supported by this study. The stigma associated with this disease might be due to a lack of knowledge and discrimination in society against those diagnosed positive. The stigma associated with the disease might lead to the non-disclosure of HIV status to a partner. Gender discrimination in which the female is abused and abandoned by her partner and his family when diagnosed with HIV holds them back from getting tested. Another study states, women are fearful about domestic violence and abandonment by their partner, which limits disclosure of their HIV status to their partner [30].

Delays from the side of healthcare professionals were another barrier identified because of the inability to assess the early symptoms, elicit the risky behaviors, and misdiagnose the condition as other illnesses. Delay from the side of the health system can also be in proper counseling by the integrated counseling and testing center (ICTC)/ART staff to remove the fear and stigma and prompt an HIV test for the partner or children of PLHIV. The absence of routine HIV testing among adults is another factor that results in late diagnosis individually, and it is interlinked with other factors, as it drops the rate of asymptomatic patients tested for HIV. Being asymptomatic and the failure of the health system to provide adequate counseling and advice, HIV testing with low health literacy may lead to a perceived good health status for the individual. It leads to a false sense of security and ignorant behavior, even when suggested by peers or other members of society, which leads to late presentation to HIV care.

Strength of the study

Triangulation is conducted at the design (sequential), method (connecting), and reporting (weaving) levels.

Limitations of the study

The major limitation of the study was the inclusion of previously diagnosed patients registered at the ART Center, All India Institute of Medical Sciences, Rishikesh, India. Conducting a prospective study only focusing on newly diagnosed patients would have provided a more comprehensive understanding of the current situation. Including only a single academic hospital might have caused selection bias and limited the generalizability of results. Perspective of healthcare professionals was not evaluated which would help to get better insight into the barriers for HIV testing.

## Conclusions

The prevalence of LP was identified as high, and the general population was found to have a higher proportion of LP than the high-risk and bridge populations. Older age, married individuals, and heterosexual contact were found to be associated with late presentation. Pulmonary tuberculosis was the most common opportunistic infection, and it was more commonly seen in LPs. Most of the barriers identified in this study linked back to a lack of awareness about HIV, which gave a false sense of security among the PLHIV. This unawareness is associated with various interlinked factors, such as the personal, social, and environmental behavior of an individual, influencing the perception of risk and need for HIV testing.

The results indicate the necessity for implementing guidelines regarding regular HIV testing among adults and adolescents in India, with a recommended frequency of at least once a year, particularly if there are any risk factors such as blood transfusion. The little awareness of HIV infection highlights the necessity for extensive social and behavioral change communication using social media platforms, stakeholder engagement, and community involvement at every stage. New initiatives for sensitizing healthcare professionals to enable them to identify the chance of HIV infection even in low-risk patients should be organized. Understanding the issues of ICTC/ART center staff and corrective measures to be taken for creating a fear-free environment for the client to get tested and initiate treatment. Additionally, prioritize counseling for PLHIV to encourage their partners or children to get tested.

## Appendices

 Appendix A

**Table 4:** Study questionnaire Credits: Drs. Athulya V Ajith and Meenakshi Khapre

Sl. No.	Topic	Response	Remarks
A.	Demographic details		
A1.	ART Registration number:		
A2.	Name:		
A3.	Age:		
A4.	Sex:		
A5.	Residence:		
A6.	Estimated monthly household income:		
A7.	economic status:		
A8.	Education:	a) Illiterate b) Primary c) Secondary d) High School e) Intermediate f) College & above	
A9.	Occupational status:		
A10.	Marital status:	a) Single b) Married c) Widowed d) Divorce/separated	
A11.	Occupation of the spouse:		
B.	Personal details		
B1.	Alcohol intake:	Yes No	
B2.	B2. Smoking:	Yes No	
B3.	Tattoo:	Yes No	
B4.	HIV status of spouse:	Yes No	Only if answer of Q.A10 is b
B5.	HIV status of spouse at death:	Yes No Unknown	Only if answer of Q.A10 is c
C.	History of illness		
C1.	Probable mode of transmission:	1. Sexual heterosexual/homosexual 2. Injection drug use 3. Blood transfusion 4. Mother to child 5. Probable unsafe injection 6. Tattooing 7. Unknown	
C2.	Type of patient:	1. General 2. ANC/breastfeeding 3. MSM 4. TS/TG 5. FSW 6. IDU 7. Migrant 8. Truck driver 9. Others	
C3.	Health care worker:	Yes No	If No, skip to C5.
C4.	If yes, Needle prick injury:	Yes No Don’t know	
C5.	At the time of diagnosis, aware of modes of transmission of HIV:	Yes No	
C6.	Date of diagnosis of HIV:		
C7.	Method of diagnosis:	1. Self-referral 2. Referred by Physician 3. Incidental diagnosis 4. Routine Antenatal check-up	
C8.	If 1 or 2 symptoms lead to diagnosis:	a) Fever b) Weight loss c) Persistent diarrhea d) Respiratory symptoms e) Dermatological symptoms f) Spouse diagnosed positive d) Others	
C9.	CD4 count:		
	Time of diagnosis:		
	Follow up ART visits (date and reported CD4 count):		
	Recent:		
C10.	At time of diagnosis, Clinical stage according to WHO:	1 2 3 4	
C11.	Comorbidities/opportunistic infections at time of diagnosis: a) present at the time of diagnosis:	a) Yes b) No	
	b) Status of treatment:	i) No treatment taken ii) On treatment currently iii) Treatment completed	
D.	Treatment details		
D1.	Date of ART initiation:		
D2.	Any discontinuation/ treatment drop:	a) Yes b) No	If No, skip to D4.
D3.	If yes, duration:		
D4.	Initial antiviral regimen:		
D5.	Current antiviral regimen:		

Appendix B 

**Table 5:** In-depth interview guide Credits: Drs. Athulya V Ajith and Meenakshi Khapre

Name of interviewer: Dr. Athulya V Ajith, Designation: Junior Resident
Topic: Proportion of Late Presenters to HIV Care and Barriers to Early Presentation: A Mixed-Methods Study in a Tertiary Care Hospital in Uttarakhand, India
Objective:
To assess the correlates of late presentation to HIV care
To assess the barriers to seeking HIV care among late presenters
Questions:
What was the reason for you to undergo HIV testing?
Were you aware of the mode of HIV infection and various high-risk behaviors associated with HIV infection when you advised the test?
How much knowledge did you have about facilities provided for HIV care by the Government?
After being advised to undergo the test for HIV or being aware of the risk, did you delay getting it tested? If so, what were the factors responsible for it?
What was the response from your partner or family members when you decided to undergo test for HIV?
What were the issues concerning when you finally decided to get it tested and how did you address them?
What were the factors that triggered you to finally get it tested?
What was the attitude and response of health care workers during the course of your diagnosis and treatment?
What are the other difficulties you are facing that you can attribute to being diagnosed with HIV and continuing the treatment?
Additional remarks:
Debrief:

## References

[1] Gale NK, Heath G, Cameron E, Rashid S, Redwood S (2013). Using the framework method for the analysis of qualitative data in multi-disciplinary health research. BMC Med Res Methodol.

[2] Bandura A (1994). Social cognitive theory and exercise of control over HIV infection. Preventing AIDS. AIDS Prevention and Mental Health.

[3] Rao S, Satheesh AV, Unnikrishnan B, Madi D, Shetty AK Correlates of late presentation to HIV care in a South Indian cohort. Am J Trop Med Hyg.

[4] Mojumdar K, Vajpayee M, Chauhan NK, Mendiratta S (2010). Late presenters to HIV care and treatment, identification of associated risk factors in HIV-1 infected Indian population. BMC Public Health.

[5] Alvarez-Uria G, Midde M, Pakam R, Kannan S, Bachu L, Naik PK (2012). Factors associated with late presentation of HIV and estimation of antiretroviral treatment need according to CD4 lymphocyte count in a resource-limited setting: data from an HIV Cohort Study in India. Interdiscip Perspect Infect Dis.

[6] Gesesew HA, Ward P, Woldemichael K, Mwanri L (2018). Late presentation for HIV care in Southwest Ethiopia in 2003-2015: prevalence, trend, outcomes and risk factors. BMC Infect Dis.

[7] Sweeney SM, Vanable PA (2016). The association of HIV-related stigma to HIV medication adherence: a systematic review and synthesis of the literature. AIDS Behav.

[8] Degno S, Atlaw D, Mekonnen A (2021). Predictors of late presentation for HIV/AIDS in West Arsi Zone public health institutions, south Ethiopia: unmatched case-control study. HIV AIDS (Auckl).

[9] Damtie D, Yismaw G, Woldeyohannes D, Anagaw B (2013). Common opportunistic infections and their CD4 cell correlates among HIV-infected patients attending at antiretroviral therapy clinic of Gondar University Hospital, Northwest Ethiopia. BMC Res Notes.

[10] Dembelu M, Woseneleh T (2021). Prevalence of and factors associated with reoccurrence of opportunistic infections among adult HIV/AIDS patients attending the ART clinic at public health facilities in Arba Minch town, southern Ethiopia. HIV AIDS (Auckl).

[11] Mayston R, Lazarus A, Patel V (2016). Pathways to HIV testing and care in Goa, India: exploring psychosocial barriers and facilitators using mixed methods. BMC Public Health.

[12] Gardner AT, Napier R, Brown B (2016). Risk factors for “late-to-test” HIV diagnosis in Riverside County, California. Medicine (Baltimore).

[13] Jeong SJ, Italiano C, Chaiwarith R (2016). Late presentation into care of HIV disease and its associated factors in Asia: results of TAHOD. AIDS Res Hum Retroviruses.

[14] Mocroft A, Lundgren J, Antinori A (2015). Late presentation for HIV care across Europe: update from the Collaboration of Observational HIV Epidemiological Research Europe (COHERE) study, 2010 to 2013. Euro Surveill.

[15] Creswell JW, Creswell JD (2018). Research Design: Qualitative, Quantitative, and Mixed Methods Approaches. https://books.google.co.in/books/about/Research_Design.html?id=s4ViswEACAAJ&redir_esc=y.

[16] Luma HN, Jua P, Donfack OT (2018). Late presentation to HIV/AIDS care at the Douala General Hospital, Cameroon: its associated factors, and consequences. BMC Infect Dis.

[17] Chone JS, Abecasis AB, Varandas L (2022). Determinants of late HIV presentation at Ndlavela health center in Mozambique. Int J Environ Res Public Health.

[18] Sepkowitz KA (2006). One disease, two epidemics - AIDS at 25. N Engl J Med.

[19] Darling KE, Hachfeld A, Cavassini M, Kirk O, Furrer H, Wandeler G (2016). Late presentation to HIV care despite good access to health services: current epidemiological trends and how to do better. Swiss Med Wkly.

[20] Ghate M, Deshpande S, Tripathy S (2009). Incidence of common opportunistic infections in HIV-infected individuals in Pune, India: analysis by stages of immunosuppression represented by CD4 counts. Int J Infect Dis.

[21] Gautam H, Bhalla P, Saini S, Uppal B, Kaur R, Baveja CP, Dewan R (2009). Epidemiology of opportunistic infections and its correlation with CD4 T-lymphocyte counts and plasma viral load among HIV-positive patients at a tertiary care hospital in India. J Int Assoc Physicians AIDS Care (Chic).

[22] Steinbrook R (2007). Tuberculosis and HIV in India. https://www.nejm.org/doi/full/10.1056/NEJMp078049.

[23] Celesia BM, Castronuovo D, Pinzone MR (2013). Late presentation of HIV infection: predictors of delayed diagnosis and survival in Eastern Sicily. Eur Rev Med Pharmacol Sci.

[24] Darcis G, Lambert I, Sauvage AS (2018). Factors associated with late presentation for HIV care in a single Belgian reference center: 2006-2017. Sci Rep.

[25] Strauss M, Rhodes B, George G (2015). A qualitative analysis of the barriers and facilitators of HIV counselling and testing perceived by adolescents in South Africa. BMC Health Serv Res.

[26] Scott SR, Wu Z (2019). Risks and challenges of HIV infection transmitted via blood transfusion. Biosafety and Health.

[27] Njagi F, Maharaj P (2006). Access to voluntary counselling and testing services: perspectives of young people. S Afr Rev Sociol.

[28] Young SD, Hlavka Z, Modiba P (2010). HIV-related stigma, social norms, and HIV testing in Soweto and Vulindlela, South Africa: National Institutes of Mental Health Project Accept (HPTN 043). J Acquir Immune Defic Syndr.

[29] MacPhail C, Pettifor A, Moyo W, Rees H (2009). Factors associated with HIV testing among sexually active South African youth aged 15-24 years. AIDS Care.

[30] Lofgren SM, Tsui S, Atuyambe L (2022). Barriers to HIV care in Uganda and implications for universal test-and-treat: a qualitative study. AIDS Care.

